# Predictors of restenosis following percutaneous coronary stent implantation: The role of trimetazidine therapy

**DOI:** 10.3389/fcvm.2022.873899

**Published:** 2022-07-22

**Authors:** Gábor Csató, Nóra Erdei, Beatrix Ványai, Tímea Balla, Dániel Czuriga, Zoltán Csanádi, Zsolt Koszegi, István Édes, Gábor Tamás Szabó

**Affiliations:** ^1^Department of Cardiology, Faculty of Medicine, University of Debrecen, Debrecen, Hungary; ^2^Department of Dermatology, Faculty of Medicine, University of Debrecen, Debrecen, Hungary

**Keywords:** in-stent restenosis, trimetazidine (TMZ), percutaneous coronary intervention (PCI), anti-inflammatory effect, stent implantation

## Abstract

**Aims:**

In-stent restenosis (ISR) is an unresolved problem following percutaneous coronary intervention (PCI), having a negative impact on clinical outcome. The main goal of this study was to find new independent predictors that may influence the development of ISR.

**Methods and results:**

In this retrospective analysis, 653 PCI patients were involved. All patients had coronary stent implantation and a follow-up coronary angiography. Based on the presence of ISR at follow-up, patients were divided into two groups: 221 in the ISR and 432 in the control group. When evaluating the medical therapy of patients, significantly more patients were on trimetazidine (TMZ) in the control compared to the ISR group (*p* = 0.039). TMZ was found to be an independent predictor of a lower degree of ISR development (*p* = 0.007). TMZ treatment was especially effective in bare metal stent (BMS)-implanted chronic coronary syndrome (CCS) patients with narrow coronary arteries. The inflammation marker neutrophil to lymphocyte ratio (NLR) was significantly elevated at baseline in the ISR group compared to controls. The reduction of post-PCI NLR was associated with improved efficacy of TMZ to prevent ISR development. Drug eluting stent implantation (*p* < 0.001) and increased stent diameter (*p* < 0.001) were the most important independent predictors of a lower degree of ISR development, while the use of longer stents (*p* = 0.005) was a major independent predictor of an increased ISR risk.

**Conclusion:**

TMZ reduces the occurrence of ISR following PCI, with special effectiveness in BMS-implanted patients having CCS and narrow coronary arteries. TMZ treatment may help to lower ISR formation in countries with high BMS utilization rates.

## Introduction

Coronary artery disease (CAD) is one of the most common cardiovascular (CV) diseases and is a leading cause of death worldwide (1). Therefore, in CAD, coronary artery revascularization by percutaneous coronary interventions (PCI) are used in an increasing number to prevent acute coronary syndrome (ACS), and to improve symptoms and prognosis. Current guidelines emphasize the optimal patient selection for PCI, as clinical data confirmed that despite a successful PCI, angina symptoms may persist (2). Moreover, recent studies and meta-analyses suggest a controversial result of PCI on the improvement of clinical prognosis (3). One reason for these contradictory results after PCI may be the development of in-stent restenosis (ISR) leading to repeat revascularization. Inflammation, extracellular matrix remodeling, smooth muscle cell proliferation and altered cellular metabolism are all responsible for the expansion of ISR (4). Despite large improvements in both stent technologies and interventional techniques, ISR remained a constant and unresolved problem of interventional cardiology. It has a negative impact on patient outcome after PCI and is regarded as an independent risk factor for mortality (5).

Trimetazidine (1-[2,3,4-trimethoxybenzyl] piperazine dihydrochloride; TMZ) represents a unique agent in cardiovascular pharmacotherapy, which primarily targets the mitochondrial energetics by reducing oxygen consumption and the formation of reactive oxygen species (6, 7). TMZ is currently used as a second-line agent in CAD for symptom reduction (2). However, there are substantial amount of clinical evidence suggesting that TMZ has positive effects on endothelial function, as well as on local inflammation cascade after stent implantation (8–15).

Since the administration of TMZ in CAD patients improves anginal symptoms (ischemia) and endothelial dysfunction, we decided to examine our database while seeking for factors, including CV drugs, that may affect ISR development. Our aim was to find novel therapy- and patient-related independent predictors potentially influencing ISR formation. To put our hypothesis to the test, we retrospectively evaluated our database for: (1) PCI procedural characteristics, (2) CV pharmacotherapy (3) demographic factors, comorbidities, and CV risk factors and (4) blood chemistry and blood count data. Our main objective was to examine the difference between patients with and without ISR with the expectation that the optimal pharmacotherapy (including TMZ) may lead to a lower degree of ISR development.

## Materials and methods

### Study population, indication for PCI

The data collection was carried out on consecutive patients undergoing PCI in our center between 1st January 2010 and 31st December 2011, if follow-up coronary angiography was also performed by any cause within 600 days. The indication for the index PCI was ACS or chronic coronary syndrome (CCS) with severe stenosis in one of the coronary arteries. ACS was diagnosed when the criteria of the universal definition of myocardial infarction were met (16). The intervention and the medical therapy of study patients were in line with the guidelines in effect on ACS and on coronary revascularization. In most patients with ACS, pre-hospital triage with transtelephonic ECG and direct referral for catheter-based therapy were used (17, 18).

The index PCI was defined as the first stent implantation during the inclusion period. The reference vessel size was ≥2.25 mm in all lesions by quantitative measurement.

### Parameters evaluated in the study

Medical records were collected and analyzed. Demographic parameters, such as age and sex, clinical characteristics including risk factors (smoking status and alcohol consumption), co-morbidities (hypertension, hyperlipidemia, diabetes mellitus and renal impairment), relevant CV conditions (prior ACS, PCI in another coronary artery, coronary artery bypass graft surgery and heart failure) and physical examination results (body mass index, resting heart rate), as well as blood test and echocardiography results were recorded. Laboratory parameters were assessed at the time of both the index and follow-up procedures. The results of transthoracic echocardiography (left ventricular ejection fraction and left ventricular wall motion abnormalities) were evaluated at baseline.

### PCI procedural data

All coronary angiography- and PCI-related data were re-analyzed and re-evaluated by two independent invasive cardiologists in terms of coronary anatomy (the dominance of coronary artery circulation: left, right, intermediate, and super-right type; and the branch and segment of the intervened coronary artery), the characteristics of the lesion: degree of the stenosis, presence of dissection, as well as the complexity of the lesion (bifurcation). Procedural circumstances of the PCI including balloon dilatation pre- or post-stent implantation, the type, length, and diameter of the implanted stents were registered. Clinically relevant ISR was defined angiographically as equal or more than 50% diameter stenosis in the stented vessel tract, or 2 mm proximal or distal to the stent edges.

The study was conducted in accordance with the Declaration of Helsinki. Study data were collected with the written consent of the patients. Data management and collection procedures were approved by the institutional review boards of the Department of Cardiology and the University of Debrecen, Hungary (Protocol code: 5903-2021).

### Statistical analysis

A statistical analysis was performed by the GB-Stat v8.0 program (Dynamic Microsystems Inc.). Normally distributed continuous variables were compared by using Student's *t*-test at an α-level of 5%; while comparative analysis of categorical variables was carried out using the Wilcoxon rank-sum test (α-level of 5%).

Predetermined variables between patients in the ISR and control groups with a *p* < 0.4 were assessed by applying a univariate logistic regression model. Odds ratios (OR) and 95% confidence intervals (CI) were calculated. In case of multiple regression, predictors of ISR development displaying a *p*-value of <0.05 in a univariate analysis were selected and quantified for adjusted odds ratios and Cis.

A *p*-value of < 0.05 was regarded as significant. Heat maps were produced for a relation analysis to illustrate the effect of TMZ on ISR development under various conditions (DES or BMS implantation, stent length, and diameter).

## Results

Altogether, 653 PCI patients were involved in the study. The mean age of the patients was 61.93 ± 10.37 years. The indication for the index PCI was ACS (341 cases) or CCS (312 cases). During index PCI, altogether 910 stents were implanted: bare metal stents (BMS) in 78.56%, drug eluting stents (DES) in 21.44% of the cases. Second generation DES was used in 96% of DES implantation (sirolimus or sirolimus derivates as active drug with the distribution as follows: everolimus: 56, zotarolimus: 43, sirolimus: 11, biolimus: 20, tacrolimus: 1, and amphilimus: (1) The average stent/subject number was 1.38 ± 0.69. The ISR rate was 33.84% in the total study population. In our patient cohort, the development of ISR was independent from the initial clinical diagnosis (ACS: 33.34%, and CCS: 34.29%).

Follow-up coronary angiography was performed within an average of 198 ± 150 days following the index PCI. The major reasons for follow-up coronary angiography were crescendo angina or staged PCI (608 cases) and ACS (45 cases) following the index PCI.

Based on the presence of clinically relevant ISR at the follow-up coronary angiography, study participants were divided into two groups: 221 patients in the ISR group and 432 patients in the control (no ISR) group. The baseline characteristics and the CV pharmacotherapy is presented in Table 1. Proportionally, significantly more females were present in the ISR group compared to controls. Interestingly, among patients with regular alcohol consumption, the ISR was also significantly more frequent. More diabetes was noted in the ISR group compared to control, but the difference between the two groups did not prove to be significant. Regarding CV risk factors and concomitant diseases, the two groups were relatively well-balanced.

**Table 1:** Baseline clinical characteristics and medical therapy.

**Parameter**	**Total patient population**	**Control—patients without ISR**	**Patients with ISR**	***p*** **-value**
	**(*****n*** = **653)**	**(*****n*** = **432)**	**(*****n*** = **221)**	
Age (y)	61.93 ± 10.37	61.59 ± 10.23	62.61 ± 10.37	0.233
Male gender (%)	62.94	65.97	57.01	0.026
Female gender (%)	37.06	34.03	42.99	
BMI > 25 (%)	34.76	33.79	36.65	0.488
Current tobacco use (%)	24.66	24.07	25.79	0.633
Current alcohol use (%)	5.21	3.47	8.60	0.008
Hypertension (%)	69.98	69.91	70.14	0.962
Hyperlipidemia (%)	87.74	89.25	84.80	0.202
Diabetes (%)	29.10	27.77	31.67	0.317
Heart failure (%)	8.58	8.10	9.50	0.557
Previous ACS (%)	30.01	30.32	29.41	0.857
Previous CABG (%)	7.96	8.33	7.24	0.760
Previous PCI (%)	27.72	28.47	26.24	0.580
Acute indication of PCI (%)	51.45	52.31	49.77	0.840
Left ventricular EF (%)*	50.46 ± 8.61	50.45 ± 8.82	50.48 ± 8.22	0.966
Pharmacotherapy				
DAPT (%)	100	100	100	1.000
Oral anticoagulants (VKA) (%)	12.10	12.96	10.41	0.377
Trimetazidine (%)	54.21	56.94	48.86	0.039
Statin (%)	94.03	95.14	91.86	0.115
ACEi and/or ARB (%)	93.72	93.67	93.75	0.898
CCA (%)	31.39	32.87	28.51	0.285
BB (%)	93.87	93.75	94.11	0.896
Insulin (%)	8.73	8.33	9.50	0.919
OAD (%)	13.78	13.43	14.48	0.349
OAD+insulin (%)	1.84	1.85	1.81	0.993

Values are means ± SD or percentages of subjects. ^*^For left ventricular EF, the numbers in the control and ISR groups were 394 and 203, respectively.

*ACS, acute coronary syndrome; ACEi, angiotensin converting enzyme inhibitor; ARB, angiotensin II receptor blocker; BB, β-receptor blocker; BMI, body mass index; CABG, coronary artery bypass graft; CCA, calcium channel antagonist; DAPT, dual antiplatelet therapy; EF, ejection fraction; ISR, in-stent restenosis; OAD, oral antidiabetics; PCI, percutaneous coronary intervention; VKA, vitamin K antagonist. In this instance, the p-value refers to differences between patient groups with and without restenosis*.

Standard of care CV pharmacotherapy was used in the study population according to guidelines in effect (Table 1). TMZ was administered in 54.21% of cases (35 mg twice daily) for symptom relief in patients with previous history of angina. There was no significant difference between the two groups regarding CV pharmacotherapy with one exception: proportionally, significantly more patients were on TMZ in the control group as compared to the ISR group (Table 1).

The PCI-related parameters are detailed in Table 2. Highly significant differences were detected between the groups regarding BMS and DES implantations. Most of the DES implantations were carried out in the control group. On the other hand, BMS implantations were much more frequent in the ISR group. Examination of the stent/patient ratio, the total length and the diameter of the implanted stents revealed significant differences between groups (Table 2). The proportion of patients with balloon dilatation before stent implantation was also slightly higher in the ISR group, compared to controls, but the difference was statistically not significant (*p* = 0.382). There were no significant differences between the groups regarding coronary anatomy (the intervened coronary artery branch and segment), lesion characteristics and the indication of the index PCI or the follow-up coronary angiography.

**Table 2:** Index PCI-related parameters in the patient populations.

	**Total patient population**	**Control—patients without ISR**	**Patients with ISR**	***p*** **-value**
	**(*****n*** = **653)**	**(*****n*** = **432)**	**(*****n*** = **221)**	
Stent/patient (mean ± SD)	1.38 ± 0.69	1.29 ± 0.59	1.55 ± 0.82	<0.0001
BMS (%)	78.56	72.45	90.50	<0.0001
DES (%)	21.44	27.55	9.50	<0.0001
Total stent lengths (mm)	33.77 ± 21.97	30.56 ± 18.25	40.06 ± 27.80	<0.0001
Stent diameters (mm)	2.88 ± 0.43	2.94 ± 0.43	2.75 ± 0.40	<0.0001
Presence of dissection (%)	2.76	3.01	2.26	0.914
Bifurcation lesion (%)	2.60	2.77	2.26	0.953
Ballon pre-dilatation (%)	63.30	64.81	69.23	0.382
Ballon post-dilatation (%)	20.03	26.39	26.69	0.826

*Values are means ± SD or percentages of subjects*.

*BMS, bare metal stent; DES, drug eluting stent; ISR, in-stent restenosis. In this instance, the p-value refers to differences between patients with and without in-stent restenosis*.

All stent-related parameters have been examined separately in both genders. A significantly smaller implanted stent diameter was found in female patients compared to males (2.76 ± 0.39 vs. 2.95 ± 0.44 mm, *p* < 0.001). Regarding other stent-related parameters (stent/patient ratio and total stent length), no significant differences were noted between females and males.

The blood chemistry and blood counts have also been examined in all patients both at baseline and at the time of the follow-up coronary angiography (Table 3). As expected, the fasting glucose level was slightly higher in the ISR group in comparison with the control group (corresponding to the higher proportion of diabetes in the ISR group), but the difference did not prove to be statistically significant. In patients with restenosis, a slightly, but significantly lower hemoglobin level and red blood cell count was observed at baseline (Table 3). At the time of follow-up intervention, the differences found at baseline did not change significantly.

**Table 3:** Baseline blood chemistry and blood count results.

**Parameter**	**Total patient population**	**Control—patients without ISR**	**Patients with ISR**	***p*** **-value**
	**(*****n*** = **653)**	**(*****n*** = **432)**	**(*****n*** = **221)**	
Glucose (mmol/L)	7.59 ± 3.42	7.47 ± 3.41	7.84 ± 3.42	0.078
Urea (mmol/L)	6.61 ± 2.85	6.67 ± 3.11	6.49 ± 2.85	0.969
Creatinine (μmol/L)	84.31 ± 45.24	85.86 ± 52.07	81.29 ± 27.39	0.442
GFR (ml/min)	77.61 ± 17.71	77.39 ± 18.18	78.04 ± 17.71	0.828
CRP (mg/L)*	9.71 ± 22.96	9.03 ± 17.87	10.94 ± 30.03	0.384
Triglycerol (mmol/L)	1.97 ± 1.54	1.95 ± 1.44	2.00 ± 1.71	0.573
HDL–C (mmol/L)	1.23 ± 0.36	1.22 ± 0.35	1.25 ± 0.38	0.439
LDL–C (mmol/L)	3.12 ± 1.19	3.14 ± 1.18	3.06 ± 1.22	0.363
Hemoglobin (g/L)	139.11 ± 13.81	140.05 ± 14.26	137.31 ± 12.73	0.006
RBC count (T/L)	4.69 ± 0.44	4.72 ± 0.45	4.64 ± 0.42	0.029
Platelet count (G/L)	235.86 ± 73.24	230.74 ± 66.80	233.98 ± 84.31	0.873
WBC total count (G/L)	8.87 ± 3.22	8.78 ± 3.27	9.04 ± 3.22	0.953
Neutrophils (%)	66.70 ± 10.11	66.05 ± 10.18	67.95 ± 9.87	0.026
Lymphocytes (%)	24.64 ± 8.68	25.21 ± 8.79	23.55 ± 8.37	0.031
Eosinophils (%)	1.93 ± 1.70	1.99 ± 1.85	1.80 ± 1.39	0.409
Basophils (%)	0.21 ± 0.12	0.22 ± 0.12	0.20 ± 0.11	0.080
Monocytes (%)	6.62 ± 2.18	6.63 ± 2.04	6.58 ± 2.42	0.567
NLR	3.41 ± 2.46	3.28 ± 2.21	3.67 ± 2.85	0.028

*Values are means ± SD or percentages of subjects*.

^*^For the CRP, the numbers in the control and ISR groups were 262 and 150, respectively.

*CRP, C-reactive protein; GFR, glomerular filtration rate; HDL–C, high density lipoprotein cholesterol; ISR, in-stent restenosis; LDL–C, low density lipoprotein cholesterol; NLR, neutrophil/lymphocyte ratio; RBC, red blood cell count; WBC, white blood cell count. In this instance, the p-value refers to differences between patients with and without in-stent restenosis*.

**Figure 1:**
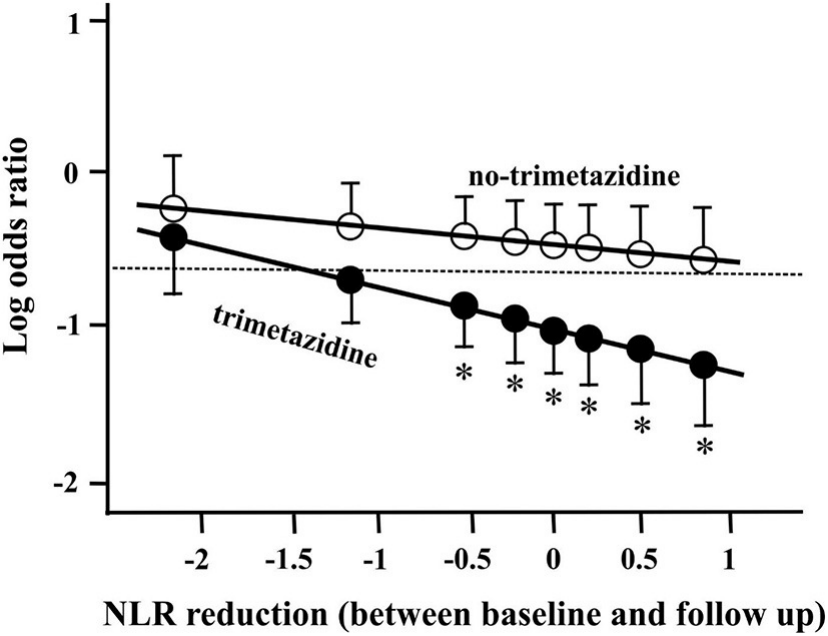
In-stent restenosis (ISR) log odds ratios and 95% confidence intervals in relation to the reduction of neutrophil lymphocyte ratio (NLR) between baseline and follow-up, defined by trimetazidine (TMZ) therapy. The dotted line represents the average ISR log odds of all patients (33.84%) in this statistical model. Asterisk (*) indicates significant difference (*p* < 0.05) between the groups (TMZ and no-TMZ).

Examination of the qualitative blood count at baseline revealed that the CV inflammation marker neutrophil to lymphocyte ratio (NLR) (19) was significantly elevated in the ISR group compared to controls (Table 3). At the time of the follow-up coronary angiography, there was a significant (*p* < 0.001) reduction in NLR in both the ISR (2.71 ± 1.09) and control (2.89 ± 1.92) groups. Interestingly, a strong relationship was noted between the magnitude of NLR reduction and ISR formation. When the ISR log ORs were presented (Figure 1) in relation to the reduction of NLR between baseline and follow-up, and defined by the TMZ therapy, then the two populations (TMZ and no-TMZ) were clearly separated. Figure 1 indicates that: (1) higher is the NLR reduction at follow-up, lower is the OR for ISR development and (2) TMZ significantly lowered the odds for ISR development at any NLR values (the relationship shifts downwards).

**Figure 2:**
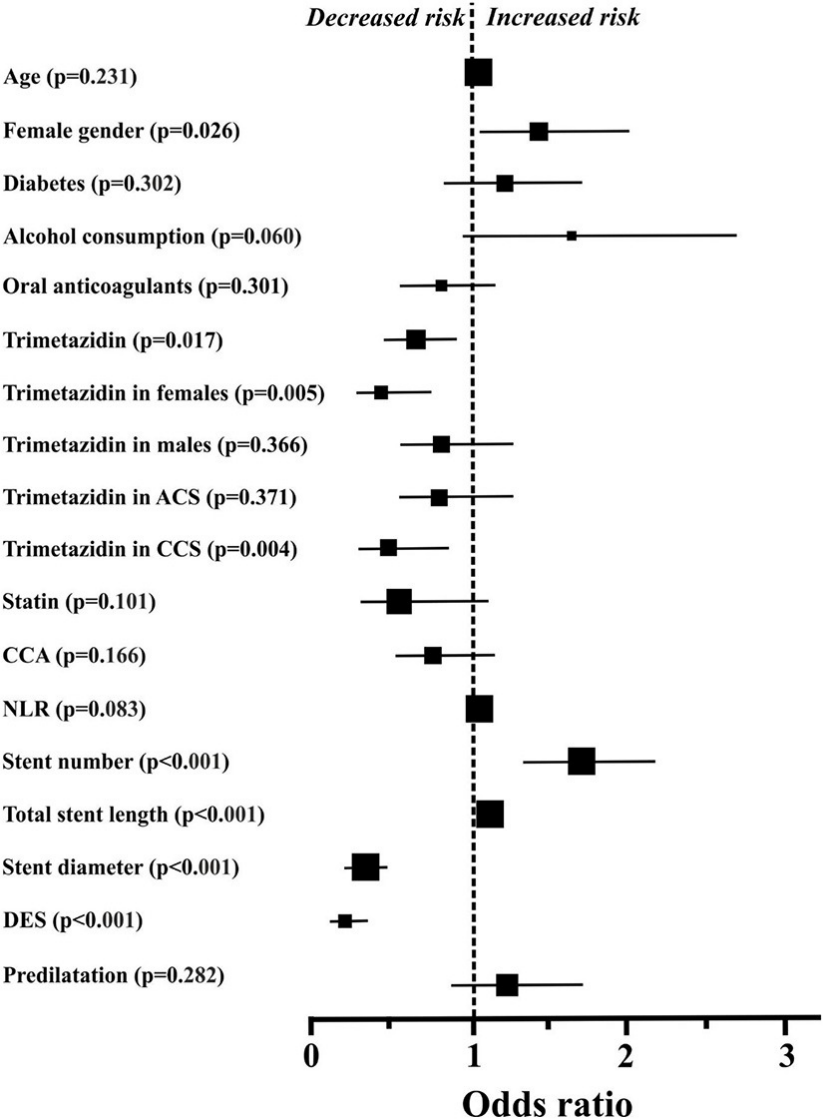
Odds ratios and 95% confidence intervals for in-stent restenosis in the individual subgroups, defined based on baseline characteristics, blood chemistry, percutaneous coronary intervention-related procedural data, and drug therapy. The sizes of the symbols reflect the number of patients in each group. For some parameters (age and total stent length), confidence intervals are within the symbols. ACS, acute coronary syndrome; CCA, calcium channel antagonist; CCS, chronic coronary syndrome; DES, drug eluting stent; NLR, neutrophil to lymphocyte ratio.

All predetermined parameters between the two groups (with *p* < 0.4) were evaluated by the univariate log-rank test (Figure 2). The ORs and CIs for ISR formation were calculated. Applying the univariate statistical method, several PCI-procedural and drug treatment-related parameters turned out to exert significant effects on ISR development. Our data analysis showed that female gender, higher stent/patient ratio and greater stent length were linked to significantly increased ORs for ISR development (Figure 2). On the other hand, DES implantation, greater stent diameter and TMZ treatment significantly lowered the ORs for ISR development (Figure 2). The effect of TMZ on ISR development was mainly due to the reduction found in female patients or patients with CCS. In fact, TMZ was not even a significant predictor for ISR development in males or in ACS (Figure 2).

Variables characterized by a *p* < 0.05 in the univariate analysis were selected for multiple regression and were quantified for adjusted ORs and CIs for ISR development. Regarding drug therapy- and PCI-related parameters, TMZ treatment (OR 0.62, CI 0.43–0.88, *p* = 0.007), DES implantation (OR 0.17, CI 0.09–0.30, *p* < 0.001) and a greater stent diameter (OR 0.33, CI 0.21–0.54, *p* < 0.001) were the most important independent predictors of decreased ISR development in our model. At the same time, the greater stent length (OR 1.02, CI 1.01–1.03, *p* = 0.005) was the major independent predictor of an increased risk of ISR development. All other parameters which were below *p* < 0.05 in the univariate analysis (i.e., stent number and gender) did not prove to be significant, independent predictors of the ISR development in our patient population.

**Figure 3:**
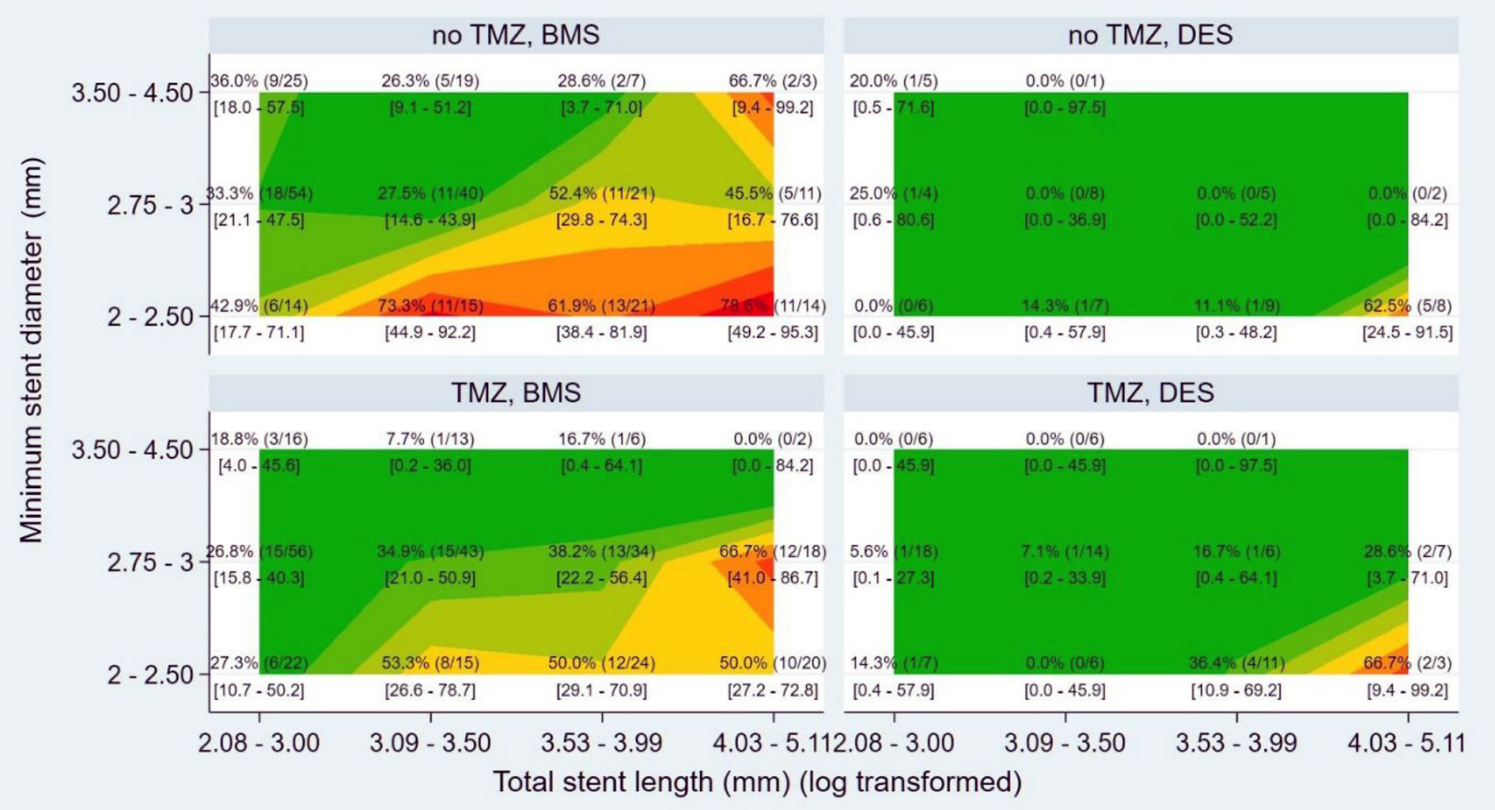
Heat map presenting the effect of trimetazidine (TMZ) in relation to stent-dependent parameters. DES, drug eluting stent; BMS, bare metal stent.

Since TMZ treatment was found to be an independent predictor of ISR development, we decided to examine the relations between TMZ treatment and the different stent parameters (type of stent, stent diameter and length) using the heat map statistical approach (Figure 3). The map clearly indicates that higher degree of ISR development was associated with (1) reduced stent diameter and (2) increased stent length both in the DES and BMS receiving patients. Moreover, the sub-analysis of the effect of TMZ to protect ISR development revealed that the efficacy of the drug is especially good on (1) narrow coronary arteries (stent diameter <3 mm, OR 0.52, CI 0.32-−0.82, *p* = 0.005), (2) BMS receiving patients (OR 0.64, CI 0.36–0.95, *p* = 0.022), and (3) CCS patients (OR 0.49, CI 0.30–0.80, *p* = 0.004).

## Discussion

The incidence of ISR widely varies in different studies and registries. In different databases, the incidence of ISR was detected up to 40% following BMS implantation (20, 21), and between 5 and 15% after DES implantation (22). In our study population, the ISR rate was found to be in the same range (BMS 38.99% and DES 15.00%).

In the development of ISR, various pathogenic mechanisms have been previously proposed. These are the (1) early elastic return (recoil), (2) vascular remodeling, and (3) neointimal hyperplasia. ISR is a non-specific inflammatory response to vessel wall injury due to a foreign element (stent) and mechanical injury during PCI. Chronic wall stress stimulates inflammatory responses and the migration of smooth muscle cells from the tunica media and tunica adventitia to the tunica intima (neointimal hyperplasia) (4, 23, 24). The introduction of DES has drastically reduced ISR development by inhibiting one of its main causes, the proliferation of the neointima. This resulted in substantial reduction in the development of ISR (25). Newer DESs are considered safer and more effective than first-generation DESs (26). Recently, the utilization of DES in any PCI has prevailed over BMS in developed countries. In accordance with these observations, we also noted in our study that DES implantation is a powerful independent predictor of lower degree ISR development.

In previous long-term follow up studies with BMS, it has been reported that minimal stent diameter <3.0 mm and total stent length >28.0 mm are independent risk factors of target vessel revascularizations including ISR (27). Similar observations were made in the present study; both the stent diameter and the total stent length have been found to be independent predictors of ISR development. It should be emphasized that BMS is still widely used in developing countries (28) and according to recent market analyses, its market share is expected to grow in the future. Consequently, all new data regarding ISR formation following BMS implantation are important.

Previously, various CV drugs, including statins (29), ACE-inhibitors (30), and calcium channel antagonists (31) were tested to prevent the development of ISR. Unfortunately, all conventional CV medications failed to reduce ISR development. In one study of an Asian population with 635 participants, however, TMZ significantly reduced ISR rates after DES implantation against placebo after 1-year follow-up (32).

In our study, a slight but significant beneficial effect of TMZ treatment on ISR development has been demonstrated. TMZ was especially effective in BMS-implanted CCS patients with narrow coronary arteries (Figures 2, 3). In our database, the rate of ISR was higher in women, as compared to men (Table 1). The role of gender regarding ISR development and the response to TMZ treatment is not completely understood yet. On the one hand, there are data indicating that DES implantation in women is associated by higher early adverse event rate including target vessel revascularization and ISR (33). Others did not reported differences between males and females in ISR and target vessel revascularization rates after DES implantation (34). Moreover, in a long-term follow-up study after BMS implantation in males, significantly more target lesion revascularizations were noted (27).

In the recent clinical trial (ATPCI), where TMZ therapy was examined in patients following PCI due to unstable angina, non-ST segment elevation myocardial infarction or CCS, no significant effects of long-term TMZ therapy were noted on the primary composite efficacy endpoint of cardiac death, hospital admission for cardiac event, or recurrent or persistent angina (35). However, in this clinical event-driven study, the ISR rate, the ISR-related adverse events or PCI procedural data were not examined in depth.

These contradictory observations are pointing to the fact that the pathophysiological background of ISR development is still not clearly understood, especially regarding the gender differences. Most clinical trials did not explore these differences in response to TMZ therapy. In our patient population, we have found that (1) stents used in females had a significantly smaller diameter compared to males and (2) TMZ treatment was significantly more effective in CCS patients with narrow coronary arteries and BMS stent implantations. Taken together, TMZ therapy may be more effective in females with CCS due to narrower coronary arteries and smaller stent diameters, especially in case of BMS implantation.

In our analysis, blood chemistry and peripheral qualitative blood results were investigated as well. At baseline, a slightly but significantly lower hemoglobin and red blood cell count were observed in patients with ISR. Moreover, the inflammatory marker NLR has also been significantly elevated in the ISR group as compared to controls. Other laboratory parameters at baseline did not differ significantly between the groups.

NLR is a novel inflammatory marker which was found to be associated with the severity and prognosis of many CV diseases (19). Inflammation is regarded as an important determinant for both the initiation and progression of CAD (36, 37). Moreover, inflammation may contribute to stent thrombosis and ISR after PCI (38–40). The relationship between NLR and angiography-defined CAD was examined in over 3,000 patients and concluded, that independently from the established CV risk factors, high NLR was associated with increased progression and complexity of coronary artery lesions (calcification and thrombus) (41). It is interesting to propose that, in accordance with previous observations, the higher NLR of our ISR study population at baseline may have contributed to the ISR formation.

In some previous studies, CRP, as an inflammatory marker has been found to be a significant predictor of ISR development in case of BMS implantation (42). In our dataset, there were no significant differences in CRP levels between the ISR and control arms. However, it should be noted that the level of CRP is depending on various factors, including statin therapy, which was administered in a high percentage among our study patients.

A recent study suggested that TMZ possesses extra-mitochondrial effects and acts as a modulator of the inflammation cascade. Specifically, it was previously shown that TMZ attenuates macrophage infiltration and pro-inflammatory responses in animal models (11). The anti-inflammatory properties of TMZ may explain, at least in part, the beneficial effects of the compound on ISR development. In accordance with these observations, we also noted that (1) baseline NLR was significantly elevated in the ISR group compared to controls and (2) TMZ shifted the relationship of ISR development vs. post-PCI NLR reduction downwardly (anti-inflammatory effect).

## Conclusion

The main goal of our present study was to find new therapy- and patient-related independent predictors that may influence the development of ISR in PCI-treated patients. In accordance with previous observations, the implantation of DES, as well as both stent diameter and total stent length have been found to be independent predictors of ISR development. Moreover, a significant beneficial effect of TMZ treatment on ISR development has been demonstrated in the overall study population. TMZ was especially effective in BMS-implanted females (narrow coronary arteries) with the diagnosis of CCS. The anti-inflammatory properties of TMZ may explain, at least in part, the beneficial effects of the compound on ISR development.

### Limitations

A potential limitation of this study is that all data analyses were performed on a retrospective basis. It should be emphasized; however, that in the data collection phase, a relatively long inclusion (2 years) was applied, and all PCI patients with follow-up angiography were included in the study database. The respective groups (ISR and control) were well-matched regarding risk factors, previous medical history, and concomitant diseases.

In our study population, predominantly BMS implantation was performed during PCI. Importantly, BMS is still widely used in developing regions. Consequently, all new data regarding ISR formation following BMS implantation are still relevant. Another limitation of our study could be that other relevant variables may not have been examined and incorporated into our model.

## Data availability statement

The original contributions presented in the study are included in the article/supplementary material, further inquiries can be directed to the corresponding author.

## Ethics statement

The studies involving human participants were reviewed and approved by University of Debrecen. The patients/participants provided their written informed consent to participate in this study.

## Author contributions

GS and IÉ: conceptualization and writing—original draft preparation. GS, DC, ZK, and IÉ: methodology. ZC, TB, BV, and NE: validation. ZK, DC, GC, ZC, and IÉ: formal analysis. GS, GC, ZK, NE, BV, and DC: investigation. GC, TB, BV, and NE: resources. GS, DC, and IÉ: data curation and visualization. IÉ, DC, and ZK: writing—review and editing. ZC, ZK, IÉ, and GS: supervision. IÉ and TB: project administration. All authors have read and agreed to the published version of the manuscript. All authors contributed to the article and approved the submitted version.

## Conflict of interest

The authors declare that the research was conducted in the absence of any commercial or financial relationships that could be construed as a potential conflict of interest.

## Publisher's note

All claims expressed in this article are solely those of the authors and do not necessarily represent those of their affiliated organizations, or those of the publisher, the editors and the reviewers. Any product that may be evaluated in this article, or claim that may be made by its manufacturer, is not guaranteed or endorsed by the publisher.
